# Case Report: Occult splenic rupture during left-sided VATS decortication: diagnostic role of early perioperative FAST

**DOI:** 10.3389/fsurg.2026.1883987

**Published:** 2026-07-20

**Authors:** Jinming Yao, Yan Xin, Xianzhen Liu

**Affiliations:** 1 Department of Anesthesia and Surgery, Qingdao Municipal Hospital, Qingdao, China; 2 Department of Anesthesia and Surgery, Qingdao Hospital of University of Health and Rehabilitation Sciences, Qingdao, China

**Keywords:** case report, early perioperative, focused assessment with sonography for trauma (FAST), splenic rupture, video-assisted thoracic surgery (VATS)

## Abstract

Video-Assisted Thoracic Surgery (VATS) is the preferred method for many thoracic diseases, featuring advantages of minimal trauma, reduced postoperative pain, and low complication rates. However, it carries potential risks, including splenic rupture—a rare but life-threatening complication. A 69-year-old male patient developed hemodynamic instability during VATS for left-sided empyema clearance; intraoperative exploration failed to identify an obvious bleeding source. Early post-procedural FAST bedside focused assessment with sonography for trauma (FAST) confirmed splenic rupture, which was managed promptly. The patient achieved full recovery and was discharged 17 days after surgery. Bedside FAST should be performed early when refractory shock occurs during VATS.

## Introduction

Compared with traditional open thoracotomy, video-assisted thoracoscopic surgery (VATS) offers advantages such as minimal trauma, reduced pain, and faster recovery, making it particularly suitable for elderly patients or those with compromised cardiopulmonary function who cannot tolerate open thoracotomy for certain pulmonary lesions, such as early-stage non-small cell lung cancer, recurrent pneumothorax with bullae, and interstitial lung disease (1, 2). Multiple clinical controlled studies and meta-analyses have demonstrated that VATS procedures, such as those for empyema clearance, are associated with a significantly lower overall incidence of perioperative complications compared to traditional open thoracotomy. This is attributed to the avoidance of extensive division of chest wall muscles and rib spreading, which substantially reduces surgical trauma and stress. Additionally, VATS results in less intraoperative blood loss and minimal interference with respiratory and circulatory functions as well as thoracic integrity (3, 4). However, splenic rupture, although relatively rare, represents a potentially life-threatening complication.

The spleen is located deep in the left upper abdomen and maintains close anatomical relationships with the diaphragm, gastric fundus, and retroperitoneum. During VATS procedures involving the left thoracic cavity, particularly procedures requiring management of the left lower basal lung segments, diaphragm, lower esophagus, or inferior mediastinum, there is a risk of inadvertent injury to the spleen and its vasculature due to traction, improper instrument manipulation, or anatomical adhesions.

Herein, we summarize a case of splenic rupture that occurred during VATS for empyema clearance in our hospital.

## Case presentation

**Figure 1:**
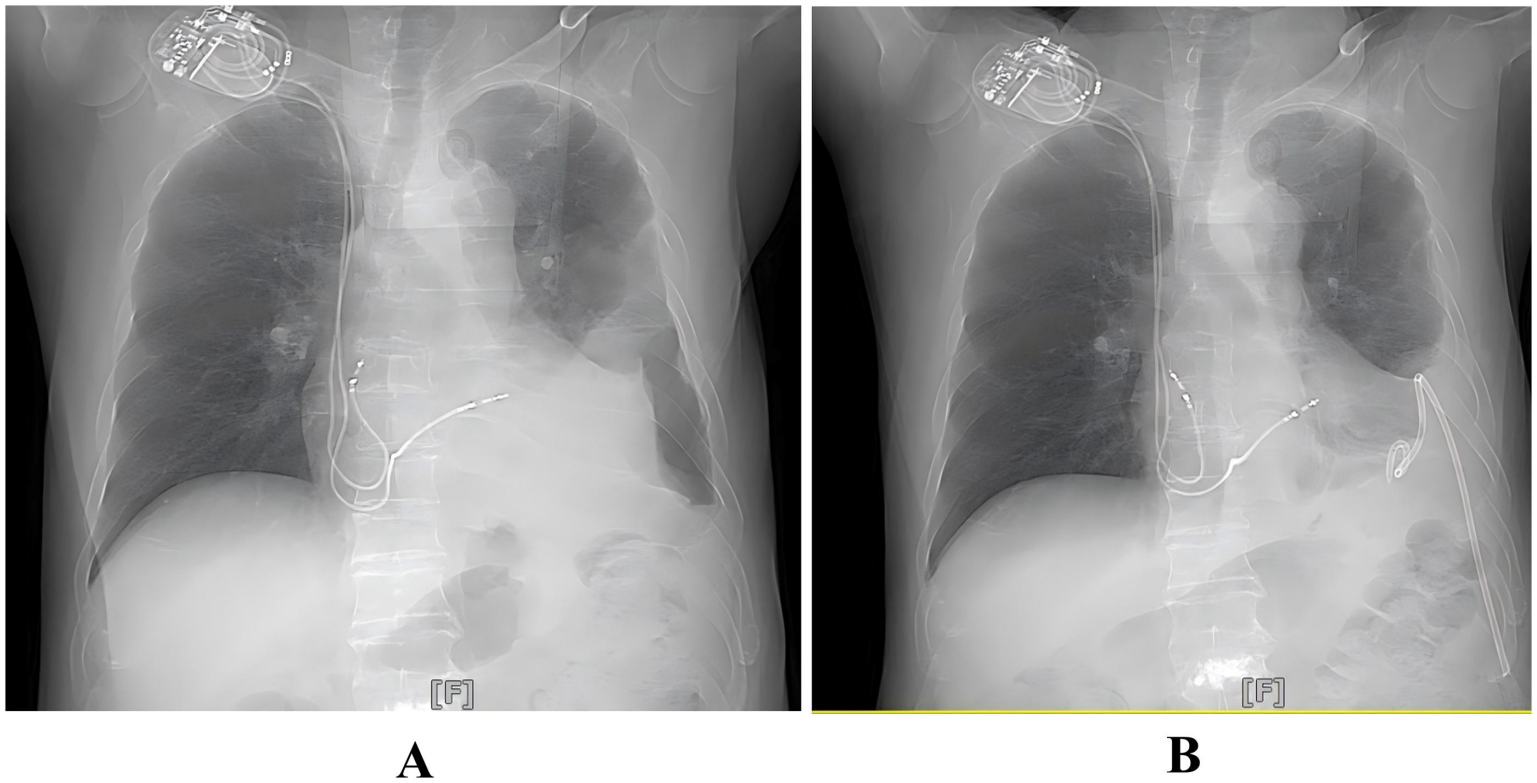
**(A)** chest CT at admission. **(B)** Chest CT after closed chest drainage.

A 69-year-old male patient (height, 175 cm; weight, 73 kg; BMI, 23.84) was admitted with an 8-day history of presumed infection and a 1-day finding of left-sided hydropneumothorax on physical examination. The patient had received anti-inflammatory treatment at a community hospital for a fever associated with the common cold. During this period, symptoms of chest tightness and dyspnea recurred, accompanied by cough, expectoration, and chest pain. On emergency admission, chest CT revealed multiple patchy nodular opacities in both lungs and left-sided hydropneumothorax with compressive atelectasis of the left lung (Figure 1A). The patient was admitted to the hospital with a left-sided hydropneumothorax and presumed empyema.

The patient had a history of well-controlled hypertension, treated with oral valsartan and amlodipine. He also had a history of poorly controlled diabetes mellitus, treated with oral dapagliflozin and acarbose, with suboptimal glycemic control (HbA1c: 9.20%). In addition, the patient had a history of coronary artery disease with prior percutaneous coronary intervention; one stent was implanted in the left anterior descending artery and another in the right coronary artery. Five years before admission, he experienced syncope due to bradycardia and underwent permanent pacemaker implantation.

Laboratory tests on admission revealed the following: complete blood count showed WBC: 25.14 × 10^9^/L, Hb: 15.2 g/dL, PLT: 317 × 10^9^/L, and CRP: 43.17 mg/L. Liver function tests indicated an albumin level of 26.72 g/L. Blood gas analysis demonstrated pH: 7.44, PO_2_: 77.1 mmHg, PCO_2_: 31.1 mmHg, Hb: 15.4 g/dL, Hct: 49.2%, K^+^: 3.78 mmol/L, Ca^2+^: 1.13 mmol/L, lactate: 2.5 mmol/L, and BE: −2.15 mmol/L. The coagulation profile and renal function were within normal limits.

Pulmonary function testing showed FVC: 2.09 L, FEV_1_: 1.5 L, FEV_1_/FVC: 72.29%, and MVV: 67.37 L/min, indicating grade III restrictive ventilatory dysfunction, along with DLCO 51%, suggesting grade II diffusion impairment. Echocardiography demonstrated a left ventricular ejection fraction of 54%, grade 1 left ventricular diastolic dysfunction, segmental ventricular wall motion abnormalities, aortic valve calcification complicated by mild stenosis and mild-to-moderate regurgitation, mild mitral and tricuspid regurgitation, as well as trace pericardial effusion. After admission, the patient received anti-infective therapy with cephalosporins and quinolones, and percutaneous thoracostomy was performed (Figure 1B). After percutaneous thoracostomy, along with blood pressure control and blood glucose stabilization, cardiopulmonary function improved, and symptoms of chest tightness and dyspnea were markedly relieved. The patient was subsequently scheduled for thoracoscopic clearance of left-sided empyema.

At induction of anesthesia in the operating room, noninvasive blood pressure (NIBP) was 127/58 mmHg, heart rate (HR) was 75 beats/min, and pulse oximetry saturation (SpO_2_) was 99%. Under ultrasound guidance, the left radial artery was cannulated for invasive pressure monitoring. Anesthesia was induced with sufentanil 30 μg, etomidate 10 mg, propofol 50 mg, and cisatracurium besylate 12 mg. A left-sided double-lumen endotracheal tube (37 Fr, depth 29 cm) was inserted, and correct positioning was confirmed by fiberoptic bronchoscopy before the tube was secured.

Anesthesia was maintained with remifentanil at 0.1–0.2 μg/(kg·min), propofol at 2–4 mg/(kg·h), inhaled sevoflurane at 1%, and intermittent intravenous boluses of cisatracurium besylate 3 mg. Surgery commenced at 13:40 with right-sided single-lung ventilation (*V_T_*: 400 mL, RR: 12 breaths/min, Paw: 20 cmH_2_O). An observation port was created through the eighth intercostal space at the left midaxillary line for thoracoscopic exploration. After exploration, an operative incision was made through the sixth intercostal space at the left anterior axillary line for thoracic entry. Thoracoscopic examination revealed empyema in the thoracic cavity. Approximately 50 mL of purulent fluid was aspirated, and the operative field was extensively covered with purulent exudate. The surgeon used an electrocoagulation hook in conjunction with atraumatic dissection forceps and a dissector to perform a combination of sharp and blunt dissection along the plane of pleural adhesions, thereby accomplishing empyema clearance.

Hemodynamics remained stable at the beginning of surgery, with invasive arterial blood pressure (ABP) of 100–120/45–55 mmHg, HR of 60–70 beats/min, and SpO_2_ of 100%. However, beginning at 14:10, ABP gradually decreased from 110/50 mmHg to a minimum of 80/40 mmHg. Ephedrine and metaraminol were administered intermittently as intravenous boluses, but hypotension recurred repeatedly. Given the severe thoracic infection and possible sepsis due to toxin absorption, fluid resuscitation was accelerated for presumed sepsis, and a norepinephrine infusion was initiated at 0.05 μg/(kg·min). At 14:32, arterial blood gas analysis revealed pH: 7.32, PO_2_: 166.3 mmHg, PCO_2_: 44.7 mmHg, Hb: 10.0 g/dL, Hct: 29%, K^+^: 3.24 mmol/L, Ca^2+^: 0.98 mmol/L, lactate: <1.0 mmol/L, and BE: −3.71 mmol/L. Hemoglobin had decreased markedly compared with the preoperative value. The thoracic surgeon was asked to explore the thoracic cavity, but no additional bleeding sites were identified. Resuscitation with crystalloid and internal milieu optimization were continued; however, blood pressure remained unstable, and HR gradually increased to 100–110 beats/min. The norepinephrine infusion rate was progressively increased from 0.05 to 0.15 μg/(kg·min). At 15:19, blood gas analysis showed a further decline in hemoglobin to 9.3 g/dL; nevertheless, comprehensive re-exploration of the left thoracic cavity still revealed no evident source of bleeding.

At 16:10, the empyema clearance procedure was completed, and the patient was repositioned to the supine position. At 16:20, the patient was fully awake with adequate recovery of spontaneous ventilation. While receiving norepinephrine at approximately 0.08 μg/(kg·min), blood pressure remained generally stable, and the double-lumen tube was removed. After extubation, the patient reported no chest tightness, dyspnea, abdominal pain, or abdominal distension or fullness. However, hemodynamics remained unstable, and continuous norepinephrine infusion was required to maintain blood pressure.

**Figure 2:**
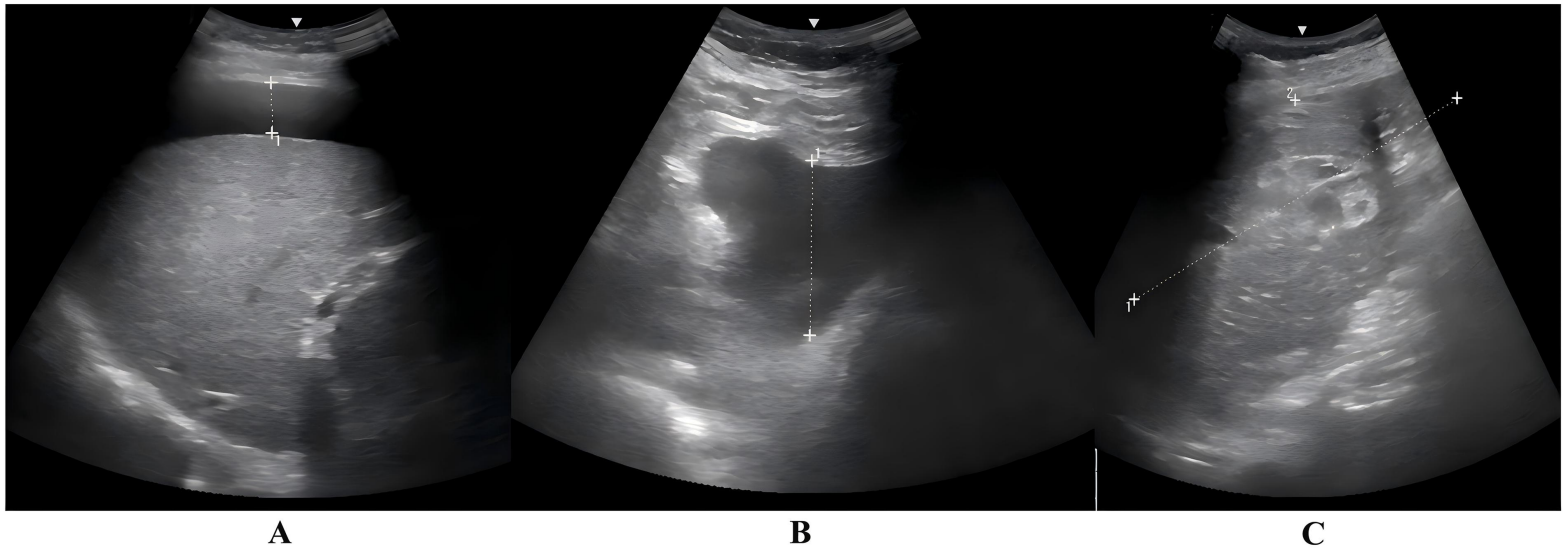
Bedside FAST exam findings. **(A)** Right upper quadrant ultrasound with free fluid surrounding the liver; **(B)** suprapubic ultrasound with free fluid in the abdominal cavity above the bladder; **(C)** left upper quadrant ultrasound with free fluid around the spleen.

At 16:34, blood gas analysis showed pH: 7.37, PO_2_: 129.7 mmHg, PCO_2_: 37.6 mmHg, Hb: 6.2 g/dL, Hct: 17.6%, K^+^: 3.70 mmol/L, Ca^2+^: 1.07 mmol/L, lactate: 1.4 mmol/L, and BE: −3.67 mmol/L. Given the substantial decrease in Hb, bedside ultrasound was performed immediately, revealing a perihepatic anechoic area with a maximum width of approximately 1.9 cm, perisplenic mixed echogenicity measuring approximately 14 cm × 4.4 cm with indistinct boundaries, and free anechoic fluid in the abdominal cavity with a maximum depth of approximately 5.6 cm (Figure 2). Diagnostic peritoneal paracentesis yielded unclotted blood.

Anesthesia was immediately induced with sufentanil 10 μg, etomidate 10 mg, and cisatracurium besylate 10 mg, followed by insertion of a 7.5# single-lumen tube. Fluid resuscitation and blood transfusion were accelerated.

**Figure 3:**
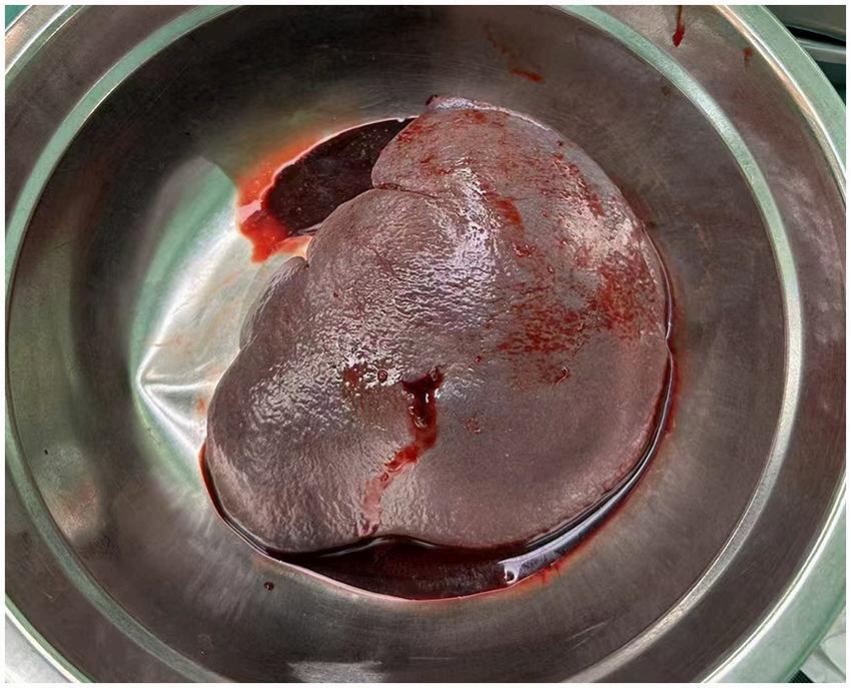
Spleen post-resection in the surgical basin with puncture visible.

Concurrently, the Department of Hepatobiliary Surgery was urgently consulted for an exploratory laparotomy, which revealed a large amount of blood clots in the left upper abdomen and a 1.5 cm × 1.2 cm laceration on the diaphragmatic surface of the mid-spleen with active bleeding (Figure 3). Splenectomy was performed. After blood transfusion, fluid resuscitation, hemodynamics gradually stabilized, with ABP: 110–130/50–60 mmHg and HR: 60–65 beats/min; norepinephrine was gradually tapered and discontinued.

At 19:35, arterial blood gas analysis showed pH: 7.31, PO_2_: 319.9 mmHg, PCO_2_: 43.4 mmHg, Hb: 8.0 g/dL, Hct: 22%, K^+^: 4.04 mmol/L, Ca^2+^: 1.05 mmol/L, lactate: 1.1 mmol/L, and BE: −4.47 mmol/L (Table 1). The operation concluded at 19:40. Ten minutes later, the patient was fully awake with adequate respiratory recovery and stable blood pressure. The endotracheal tube was removed, and the patient was transferred to the ICU for observation.

**Table 1:** Arterial blood gas and electrolyte analyses.

Parameter	Date and Timepoint					
	1/23 12:31	1/29 14:32	1/29 15:19	1/29 16:34	1/29 17:56	1/29 19:35
pH	7.44	7.32	7.30	7.37	7.22	7.31
pO_2_ (mmHg)	77.1	166.3	132.5	129.7	308.5	319.9
pCO_2_ (mmHg)	31.1	44.7	46.8	37.6	52.8	43.4
sO_2_%	96.5	99.8	99.5	100.0	100.0	100.0
Hb (g/dL)	15.4	10.0	9.3	6.2	5.8	8.0
Hct (%)	49.2	29.0	27.0	17.6	16.0	22.0
K^+^ (mmol/L)	3.78	3.24	3.66	3.70	3.98	4.04
Ca^+^ (mmol/L)	1.13	0.98	1.18	1.07	1.10	1.05
Na^+^ (mmol/L)	134.8	140.5	140.7	137.9	137.8	139.7
Cl^−^ (mmol/L)	108.4	115.6	115.8	117.6	117.0	116.1
Glu (mmol/L)	15.8	7.2	9.0	12.5	12.7	10.6
Lac (mmol/L)	2.5	<1.0	<1.0	1.4	2.3	1.1
HCO_3_^−^ (mmol/L)	20.8	22.3	22.5	21.3	21.3	21.4
HCO_3_^−^ std (mmol/L)	22.6	21.2	21.1	21.3	19.3	20.6
BE (mmol/L)	−2.15	−3.71	−3.83	−3.67	−5.85	−4.47

Total operative duration was approximately 6 h, with an estimated intraoperative blood loss of approximately 3,400 mL and urine output of 500 mL. Transfusions included 8 units of red blood cells, 800 mL of plasma, 500 mL of autologous blood, 800 mL of hydroxyethyl starch, and 2,000 mL of crystalloid solution. On postoperative day 1, the patient was transferred out of the ICU. After antibiotics administration, respiratory function training, and nutritional support, the patient recovered fully and was discharged on postoperative day 17.

## Discussion

Splenic rupture is a rare yet serious complication in thoracic surgery. When the diaphragm remains intact, hemorrhage is confined to the abdominal cavity, making intraoperative detection difficult. The few cases reported in the literature share similarities with the present case, in which no diaphragmatic rupture occurred. In these instances, blunt injury to the diaphragm affected the spleen, resulting in intra-abdominal bleeding and hemodynamic instability, with the diagnosis ultimately confirmed by ultrasound or abdominal computed tomography (5–7).

In this case, preoperative laboratory tests showed elevated white blood cell count and C-reactive protein levels. During surgery, extensive dense adhesions were observed throughout the thoracic cavity, with approximately 50 mL of encapsulated yellowish-white, thick, turbid purulent fluid. Blood pressure began to decline when the surgeon separated the pleural adhesions. Consequently, hypotension was initially attributed to sepsis, prompting rapid fluid resuscitation and infusion of vasoactive medications. However, intraoperative arterial blood gas analysis showed a progressive decline in hemoglobin, inconsistent with a diagnosis of sepsis. The surgeon repeatedly explored the thoracic cavity, but the operative field showed only oozing related to extensive adhesions, and no other bleeding foci were identified. The diaphragm remained intact, and the hemorrhage was concealed, delaying recognition of hemorrhagic shock. The patient was an elderly male with a large abdominal girth and reported no abdominal pain or distension after extubation following empyema clearance, which further confounded the diagnosis.

Accurate identification of the shock type is critical for effective management. In this case, early post-procedural bedside focused assessment with sonography for trauma (FAST) played a pivotal role in establishing the definitive diagnosis. Ultrasound is portable and noninvasive, enabling rapid assessment of potential bleeding in the pericardial, abdominal, and pelvic cavities by performing an initial four-view FAST scan, including the pericardial window, right upper quadrant (RUQ), left upper quadrant (LUQ), and suprapubic window to detect free fluid in the pericardial space, hepatorenal recess, splenorenal recess, and rectovesical pouch. This approach is particularly applicable in patients with hemodynamic instability who cannot undergo computed tomography (8).

Preoperative chest computed tomography in this patient demonstrated left-sided hydropneumothorax and atelectasis of the lingular segment of the left upper lobe and the left lower lobe. The first observation port was introduced without direct visualization through the eighth intercostal space at the left midaxillary line. We retrospectively analyzed this patient's clinical course, and persistent intrathoracic inflammation led to extensive fibrin deposition on the visceral, parietal, and diaphragmatic pleurae, followed by the formation of severe pleural adhesions. Continuous traction from widespread adhesions pulls the left hemidiaphragm cephalad, distorting its normal anatomy. Meanwhile, intraoperative one-lung ventilation induces complete collapse of the ipsilateral lung, eliminates physiological intrathoracic negative pressure, and further elevates the diaphragmatic dome. Cephalad displacement of the diaphragm shortens the anatomical distance between its peritoneal aspect and the splenic capsule, making the vulnerable splenic parenchyma prone to indirect mechanical force conduction via diaphragmatic muscles. Furthermore, blind trocar placement at the eighth intercostal space of the left midaxillary line produces transient compressive stress on the thoracic surface of the diaphragm, and sharp dissection of dense pleural adhesions brings sustained traction to diaphragmatic tissues. Although mechanical forces alone cannot rupture the intact diaphragmatic muscle layer, the transmitted kinetic energy can damage the adjacent spleen and, over time, lead to splenic injury (9). A recent multicenter study published in the Turkish Journal of Thoracic and Cardiovascular Surgery highlighted that an anatomically intact diaphragm does not constitute an absolute protective partition for thoracoabdominal viscera; biomechanical force transmission across an unruptured diaphragm accounts for 18.7% of all occult thoracoabdominal combined injuries without diaphragmatic tear (10).

Although thoracoscopic surgery has been widely applied, close vigilance against various severe complications is still required. The differential diagnosis of intraoperative hypotension during thoracic surgery for infectious lesions encompasses etiologies of circulatory, surgical, anesthetic, and cardiac origins, including septic shock caused by infection-induced systemic vasodilation, acute intrathoracic hemorrhage, occult intra-abdominal bleeding, multiple specific anesthetic triggers, and cardiogenic shock precipitated by underlying cardiac diseases such as coronary artery disease and heart failure. Specifically, anesthetic-related factors include vasodilation induced by excessive anesthetic administration, inadequate intraoperative fluid resuscitation, and reduced venous return secondary to mechanical positive-pressure ventilation.

For patients presenting with unexplained hypotension, elevated airway pressure, suspected occult hemorrhage, or hemodynamic instability, emergent bedside FAST should be performed immediately to rapidly screen for tension lesions, active bleeding, or cardiac tamponade if exhaustive thoracic exploration and serial laboratory monitoring fail to identify the underlying cause of refractory hypotension. Early perioperative FAST examination enables efficient differential diagnosis, compensates for visual blind zones resulting from diaphragmatic anatomical distortion during thoracoscopy, and addresses the inherent limitation that thoracic surgeons lack direct visualization of intra-abdominal viscera. This modality shortens the interval from shock onset to definitive diagnosis and prevents irreversible end-organ hypoperfusion injury secondary to delayed hemostasis. Flores conducted a retrospective analysis of 633 thoracoscopic lobectomy procedures, among which 12 patients experienced catastrophic complications, including several cases of major vascular injury, one case of tracheoesophageal fistula, and one case of splenic injury requiring splenectomy (11). In addition, splenic rupture related to VATS is prone to being overlooked or misdiagnosed. Liu et al. reported a case of left lower lobectomy performed by VATS in which splenic rupture occurred intraoperatively. Because the diaphragm remained intact and no bleeding was observed in the thoracic cavity, the cause of hemodynamic instability was initially attributed to cardiogenic or anaphylactic shock, and the diagnosis was ultimately confirmed by abdominal CT (7). Lateral decubitus positioning for thoracoscopic procedures substantially increases the technical difficulty of intraoperative point-of-care ultrasound assessment. It may even render this examination unfeasible in some cases. Nevertheless, this ultrasound evaluation can be performed before the patient leaves the operating room and repeated promptly if the patient's hemodynamic status deteriorates.

## Conclusion

When hemodynamic instability and a progressive decline in hemoglobin occur during VATS procedures, the possibility of subdiaphragmatic abdominal organ injury should be considered. Early perioperative FAST ultrasonography helps clarify the pathogenesis of shock and expedites rapid diagnosis and therapeutic management.

## Data Availability

The original contributions presented in the study are included in the article/Supplementary Material, further inquiries can be directed to the corresponding author.
